# Associations between *β*-Blocker Therapy at Discharge and Long-Term Follow-Up Outcomes in Patients with Unstable Angina Pectoris

**DOI:** 10.1155/2022/5287566

**Published:** 2022-09-29

**Authors:** Lei Liu, Xiaosong Ding, Hui Chen, Weiping Li, Hongwei Li

**Affiliations:** ^1^Department of Cardiology, Cardiovascular Center, Beijing Friendship Hospital, Capital Medical University, 95 Yongan Road, Beijing 100050, China; ^2^Beijing Key Laboratory of Metabolic Disorder Related Cardiovascular Disease, Beijing, China; ^3^Department of Geriatrics, Cardiovascular Center, Beijing Friendship Hospital, Capital Medical University, Beijing, China

## Abstract

**Background:**

The effects of *β*-blockers in patients with unstable angina pectoris (UAP) are unclear. We tried to evaluate associations between *β*-blockers in UAP and long-term outcomes.

**Methods:**

We enrolled 5591 UAP patients and divided them into 2 groups based on *β*-blockers at discharge: 3790 did *β*-blockers and 1801 did not used them. Propensity score matching at 1 : 1 was performed to select 1786 patients from each group. The primary endpoint was major adverse cardiac and cerebral events (MACCE) during the long-term follow-up period.

**Results:**

67.8% of patients were on *β*-blockers at discharge; these patients were more likely to have CHD risk factors, lower ejection fraction, and severity of the coronary artery lesions. Over a median of 25.0 years, the incidence of MACCE was 25.5%. The risk was not significantly different between those on and those not on *β*-blocker treatment. The multivariate Cox regression analysis showed that no *β*-blocker use at discharge was not an independent risk factor for MACCE and sequence secondary endpoints. After propensity score matching, the results were similar.

**Conclusions:**

*β*-blocker use was not associated with lower MACCE and other secondary composite endpoints in long-term outcomes. This result adds to the increasing body of evidence that the routine prescription of *β*-blockers might not be indicated in patients with UAP. Trial registration had retrospectively registered.

## 1. Introduction

Cardiovascular disease is the leading cause of death worldwide [1, 2]. *β*-Blockers have historically been integral to cardiovascular (CV) risk modification, and while the evidence for their use is most robust in patients with myocardial infarction, the evidence of effectiveness and safety mainly comes from patients with an acute myocardial fraction (AMI) and heart failure (HF), especially before the development of percutaneous coronary intervention (PCI) [3–6]. Current guidelines recommend the use of *β*-blockers in patients with coronary heart disease (CHD) early [7–9]. Whether all patients with CHD need *β*-blockers still lacks research evidence. Unstable angina pectoris (UAP), an important type of CHD, is a clinical syndrome caused by acute myocardial ischemia and accounted for a large proportion all over the world. Nowadays, the efficacy, safety, and long-term outcomes of *β*-blockers are lacking in large-scale evidence and long-term follow-up. The aim of this study, therefore, was to assess whether the use of *β*-blockers influences the incidence of major adverse cardiac and cerebral events (MACCE) in patients with UAP.

## 2. Methods

### 2.1. Study Population

**Figure 1:**
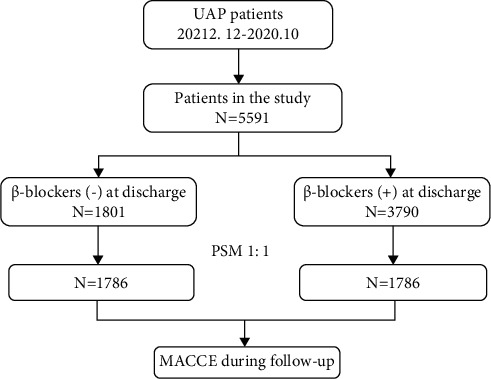
Flow chart of patient inclusion. UAP, unstable angina pectoris; MACCE, major adverse cardiac and cerebral events.

Study subjects were identified from the database at the Cardiovascular Center of Beijing Friendship Hospital. From December 2012 to October 2020, a total of 10377 consecutive patients with UAP were enrolled in this study. UAP was diagnosed based on the diagnostic criteria recommended by the European Society of Cardiology (ESC) [10]. All of them underwent coronary angiography (CAG), and the coronary stenosis was more than 70%. Exclusion criteria were as follows: (1) lacking clinical or follow-up data; (2) in-hospital death or AMI (including acute ST-segment elevation myocardial infarction and acute non-ST-segment elevation myocardial infarction); (3) infectious diseases (tuberculosis, active infective endocarditis), rheumatic disease (systemic lupus erythematosus, rheumatoid arthritis, vasculitis), hematological diseases (leukemia, lymphoma, disseminated intravascular coagulation), and neoplastic disease; and (4) contraindication of *β*-blockers such as the systolic blood pressure (SBP) at admission <90 mmHg, heart rate < 50 bpm, second-degree type II or third-degree atrioventricular block or bronchial asthma, and sick sinus syndrome. Finally, 5591 patients were included in this analysis. Based on the *β*-blockers at discharge, patients were divided into 2 groups. 1801 were not treated with *β*-blockers at discharge (*β*-blockers (-)); 3790 were confirmed to receive *β*-blocker treatment at discharge (*β*-blockers (+)). All patients were followed up on January 31, 2021, with a median follow-up of 25.0 months (IQR: 12.3, 49.2 months). The study was also designed using propensity score matching to assemble a balanced cohort. The patient flow of the study is shown in Figure 1.

The local institutional review board at our hospital approved the study protocol, and this study was in accordance with the Declaration of Helsinki.

### 2.2. Data Collection and Definitions

Patient demographic information, medical and medication history, and laboratory measurements were collected and confirmed through electronic medical records. The left atrium (LA), left ventricular end-diastolic dimension (LVEDD), left ventricular end-systolic dimension (LVESD), left ventricular ejection fraction (LVEF), and left ventricular fraction shortening (LVFS) were determined using 2-dimensional echocardiography during the index hospitalization.

The primary endpoint was major adverse cardiac and cerebral events (MACCE), which included all-cause death, heart failure (HF), nonfatal MI, nonfatal stroke, and cardiac rehospitalization at the clinical follow-up period. HF was defined as HF requiring hospital admission. Nonfatal MI was defined as chest pain with new ST-segment changes and elevation of myocardial necrosis markers to at least twice the upper limit of the normal range. Nonfatal stroke, including ischemic and hemorrhagic stroke, was defined as cerebral dysfunction caused by cerebral vascular obstruction or sudden rupture and was diagnosed based on signs of neurological dysfunction or evidence of brain imaging. Cardiac rehospitalization is referred to rehospitalization for angina pectoris or HF. In addition, to analyze mortality in more detail, cardiac death was also assessed. Cardiac death included death as a result of cardiogenic shock, MI, primary cardiac arrest, or HF. Secondary endpoints included the following: all-cause death; composite of all-cause death and HF; composite of all-cause death, HF, and nonfatal MI; and the composite of death, HF, nonfatal MI, and nonfatal stroke.

### 2.3. Statistical Analysis

Depending on the distribution of the data, continuous variables were expressed as mean value ± SD or median and interquartile range (IQR). Frequencies and percentages were used to describe categorical data. Differences between continuous and categorical variables were assessed using Student's *t*-test, analysis of variance, chi-square test, and Wilcoxon signed rank test as appropriate. In this observational study, we performed propensity score matching to reduce the effectiveness of treatment selection bias and potential confounding factors. The cumulative incidence of follow-up time MACCE was estimated by the Kaplan–Meier curves, and the groups were compared using the log-rank test.

The propensity score matching was used to reduce selection bias and confounding factors in this study. The matching process was conducted with a minimum distance scoring method and a 1-to-1 match between those on and those not on *β*-blocker treatment. The propensity score estimated the probability that patients would have been assigned to the use of *β*-blockers and was derived using a logistic regression model that included the use of *β*-blockers as the outcome variable and the following variables as predictors: age, sex, body mass index (BMI), fasting blood glucose (FBG), creatinine, alanine aminotransferase (ALT), glycosylated hemoglobin (HbA_1_C), platelet count, LA, history of CHD, old myocardial infarction (OMI), hypertension, diabetes mellitus (DM), PCI and coronary artery bypass grafting (CABG), and previous medication history of the antiplatelet agent. Ultimately, 1786 patients without *β*-blockers were individually 1 : 1 matched to 1786 patients with *β*-blocker treatment at discharge. The multivariate Cox proportional hazards regression analysis was used to assess the association between adverse clinical events and those on and those not on *β*-blocker treatment.

All analyses were two-tailed, and *P* value <0.05 was considered statistically significant. Data were analyzed using SPSS statistical package version 26.0 (SPSS Inc., Chicago, IL, USA).

## 3. Results

### 3.1. Baseline Characteristics

As shown in Table 1, of the 5591 eligible patients, 3790 patients (67.8%) used *β*-blockers at discharge and 1801 (32.2%) did not use them. Compared with the no *β*-blocker group, the *β*-blocker group showed significantly younger, higher BMI and diastolic blood pressure, higher heart rate, a higher percent of hypertension, DM, CHD, OMI, PCI, and CABG and was more likely to receive antiplatelet therapy or *β*-blockers before the hospital admission.

As presented in Table 2, the *β*-blocker group had significantly higher white cell count, platelet count, and higher levels of sensitivity C-reactive protein (hsCRP), FBG, HbA_1_C%, ALT, and triglyceride at admission than the no *β*-blocker group. Echo evaluation showed that the *β*-blocker group had significantly lower LVEF and LVFS than the no *β*-blocker group. Angiographically, the *β*-blocker group had a significantly higher percentage of multivessels, chronic total occlusions (CTO), and PCI during hospitalization. In the medication at discharge, the no *β*-blocker group had significantly more likely to receive antiplatelet therapy, ACEI/ARB, or statins.

**Figure 2:**
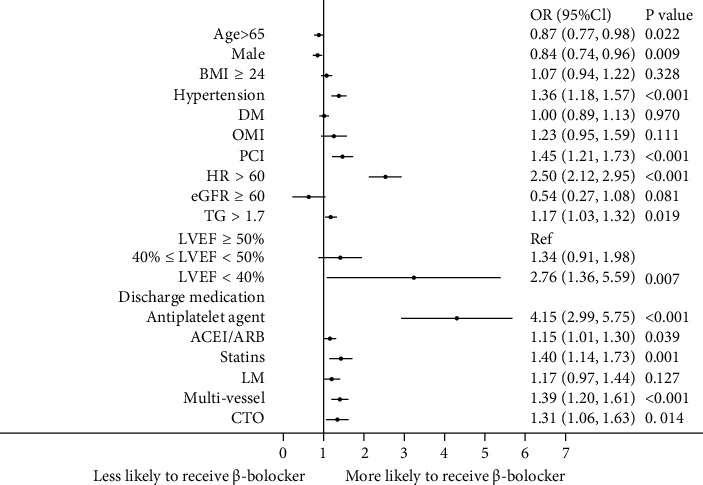
Factors associated with *β*-blocker use in multivariable analysis. Variables associated with *β*-blocker use are shown along the vertical axis. The strength of effect is shown along the horizontal axis with the vertical line demarcating an odds ratio (OR) of 1 (i.e., no association); estimates to the right (i.e., >1) are associated with a greater likelihood of *β*-blocker use, whereas those to the left (i.e., <1) indicate a reduced likelihood of *β*-blocker use. Each dot represents the point estimate of the effect of that variable in the model, whereas the line shows the 95% confidence interval (CI). BMI, body mass index; DM, diabetes mellitus; OMI, old myocardial infarction; PCI, percutaneous coronary intervention; HR, heart rate; eGFR, estimated glomerular filtration rate; TG, triglyceride; LVEF, left ventricular ejection fraction; LM, left main trunk; CTO, chronic total occlusions; ACEI, angiotensin-converting enzyme inhibitor; ARB, angiotensin II receptor blocker.

Significant correlates of *β*-blocker therapy in the multivariable analysis are shown in Figure 2. Compared with no *β*-blockers treated patients at discharge, patients prescribed *β*-blockers were more likely to be women, aged < 65 years, and had worse baseline clinical: heart rate > 60 bpm, triglyceride > 1.7 mmol/l, and lower LVEF; what is more, the proportion of hypertension, previous PCI, multivessels, and CTO is higher. Also, the *β*-blockers treated patients were more likely to receive antiplatelet or statins therapy at discharge.

### 3.2. Propensity Score Matching

Propensity scores for *β*-blocker treatment were calculated for 3572 patients, and 1786 *β*-blocker users were 1 : 1 matched to 1786 patients without using *β*-blockers at discharge. As shown in Tables 1 and 2, compared with the no *β*-blocker group, the *β*-blocker group showed a significantly higher heart rate at admission, higher levels of white cell count, hsCRP, lower LVEF, and more likely to receive *β*-blockers before hospital admission. Meanwhile, angiographically, the *β*-blocker group had a significantly higher percentage of multivessels, CTO, and PCI during CAG. In the medication at discharge, the *β*-blocker group had significantly more likely to receive antiplatelet therapy, ACEI/ARB, or statins.

There were no significant differences in baseline clinical and past medical history between the *β*-blockers and no *β*-blockers used patients for the propensity score-matched subjects.

### 3.3. Primary and Secondary Outcomes

The median follow-up period was 25.0 months (IQR: 12.3, 49.2 months). Composite MACCE occurred in 1425 patients (25.5%) in the overall population. In the *β*-blocker group, the incidence rate of composite MACCE was higher than that in the no *β*-blocker group (26.5% vs. 23.3%, *P*=0.010, Table 3). We also analyzed the event rate in the subgroups. As shown in Table 3, all-cause death occurred in 2.7% of patients in the no *β*-blocker group and 3.7% in the *β*-blocker group (*P*=0.048), and HF occurred in 1.4% of patients in the no *β*-blocker group and 2.2% in the *β*-blocker group (*P*=0.048), respectively. There was no significant difference in cardiac death, nonfatal MI, nonfatal stroke, and cardiac rehospitalization.

The univariate Cox proportional hazards regression analysis showed that there was no difference significantly between those on and not on *β*-blockers in the all-cause death, HF, nonfatal MI, nonfatal stroke, and cardiac rehospitalization groups. In addition, the adjusted hazard ratios (HRs) for composite MACCE, all-cause mortality, HF, nonfatal MI, nonfatal stroke, and cardiac rehospitalization also had no significant difference in those on *β*-blockers and those not on *β*-blockers (*P* > 0.05). The multivariate Cox proportional hazards regression analysis showed that both the *β*-blocker patients and no *β*-blockers treated patients had a similar risk of composite MACCE, all-cause mortality, HF, nonfatal MI, nonfatal stroke, or cardiac rehospitalization.

To further verify these results, we performed a sensitivity analysis using propensity score matching. These results did not change before or after further adjustment; all-cause death and the incidence of HF, nonfatal MI, nonfatal stroke, and cardiac rehospitalization in patients were similar in those on *β*-blockers and those not on *β*-blockers (Table 3).

We also analyzed the secondary endpoints. The incidence of all-cause death/HF in the *β*-blocker group was higher than that in the no *β*-blocker group (*P*=0.003), and the univariate Cox proportional hazards regression analysis showed that using *β*-blockers was a risk factor for all-cause death/HF (*P*=0.003), but after multivariate Cox proportional hazards regression analysis, the role of *β*-blockers disappeared (*P*=0.296). Similarly, in the groups of all-cause death/HF/nonfatal MI and all-cause death/HF/nonfatal MI/nonfatal stroke, the event incidences were also higher in the *β*-blockers used patients than that in no *β*-blockers (7.2% vs. 5.2%, *P*=0.006; 8.0% vs. 6.0%, *P*=0.006). *β*-blockers were risk factors in univariate Cox proportional hazards regression analysis (all *P* < 0.05), whereas these disappeared after multivariate Cox proportional hazards regression analysis.

After propensity score matching, the incidence of secondary outcomes of matched patients in the *β*-blocker group was also higher, and *β*-blockers were risk factors in the group of all-cause death/HF and all-cause death/HF/nonfatal MI, but there were also no significant differences observed after multivariate Cox proportional hazards regression analysis between the two groups (Table 4).

### 3.4. Survival

**Figure 3:**
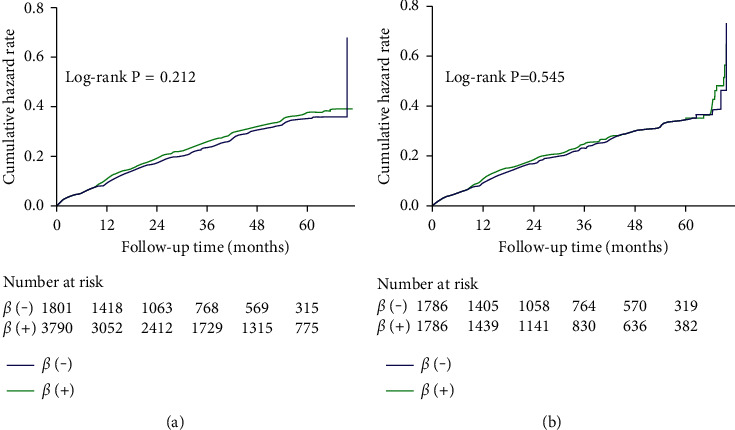
Kaplan–Meier curve for MACCE before (a) and after (b) propensity score matching patients in the *β*-blocker and no *β*-blocker groups. MACCE, major adverse cardiac and cerebral events.

In survival analysis, composite MACCE was no significant differences between the two groups. After adjusting for baseline clinical and propensity scores, there were also no significant differences (Figure 3).

**Figure 4:**
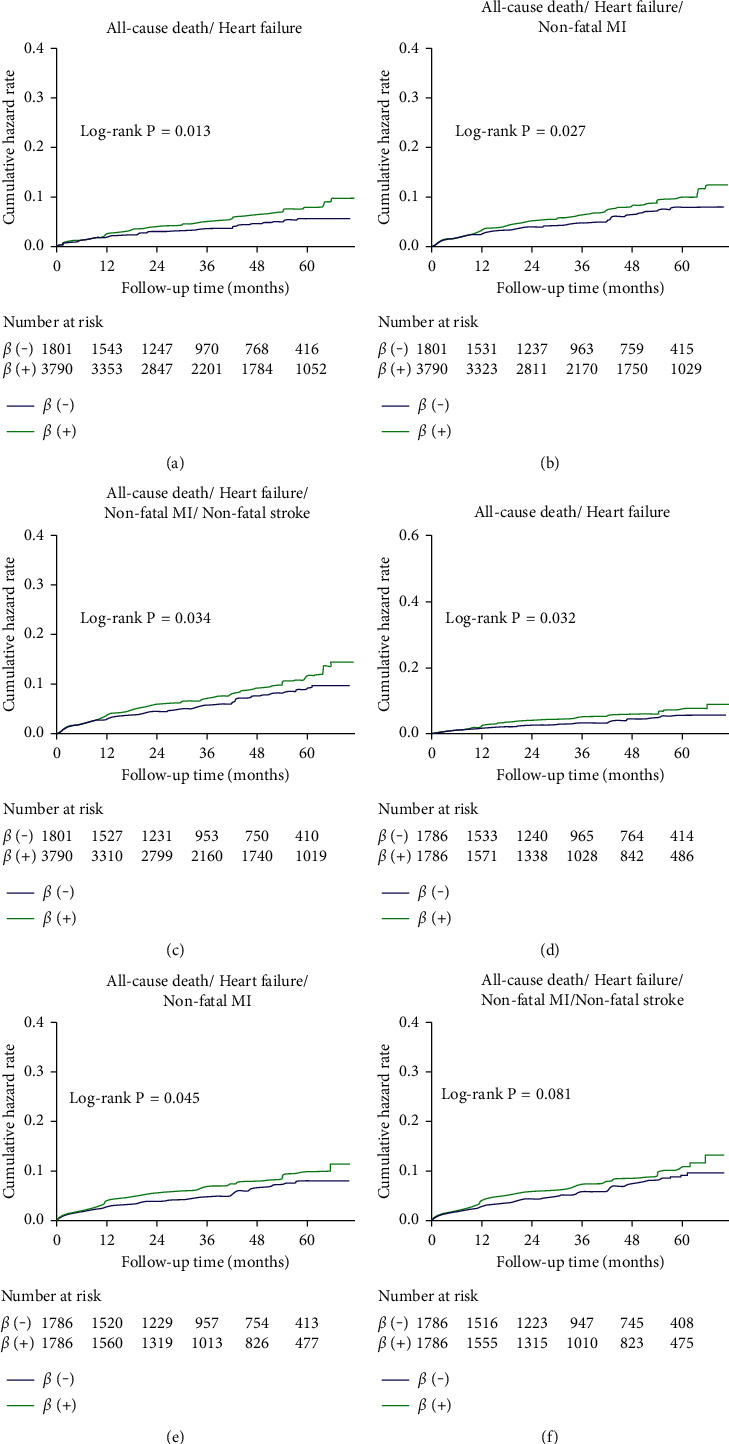
Kaplan-Meier curve for secondary endpoints before (a, b, c) and after (d, e, f) propensity score matching patients in the *β*-blocker and the no β-blocker group.

Figure 4 showed the Kaplan–Meier curves for the secondary endpoints at 25.0 months (IQR: 12.3, 49.2 months) of median follow-up period. In the outcome of all-cause death/HF, the *β*-blocker group had a significantly higher incidence than the no *β*-blocker group (*P*=0.013). In terms of all-cause death/HF/nonfatal MI and all-cause death/HF/nonfatal MI/nonfatal stroke, the *β*-blocker group also had a significantly higher incidence than the no *β*-blocker group, respectively (*P*=0.027; *P*=0.034). After adjusting for baseline clinical and propensity scores, the incidence of all-cause death/HF and all-cause death/HF/nonfatal MI was also higher in the *β*-blocker patients than in no *β*-blocker patients (*P*=0.032; *P*=0.045). However, after propensity score matching, the incidence of all-cause death/HF/nonfatal MI/nonfatal stroke was not statistically different between the two groups (*P*=0.081).

Before or after propensity score matching, the incidence of all-cause death was not statistically different between the two groups.

### 3.5. Independent Association between *β*-Blockers and Endpoints

In the multivariate analysis, we included variables that were identified to be significantly associated with secondary endpoints and composite MACCE in the univariate model. The multivariate analysis revealed that *β*-blocker therapy at discharge was not associated with primary and secondary endpoints (Tables 5 and 6); age, previous history of stroke, multivessels, left main trunk (LM), lower LVEF, higher HbA_1_C, hsCRP, and heart rate at admission were significantly and independently associated with the endpoints of all-cause death/HF and all-cause death/HF/nonfatal MI (Table 5). In terms of the secondary endpoint of all-cause death/HF/nonfatal MI/nonfatal stroke, age, previous history of stroke, multivessels, CTO, lower LVEF higher HbA_1_C, and hsCRP were the independent risk factors. With LVEF that decreased, the incidence rate of the three secondary endpoints increased correspondingly.

As shown in Table 6, after propensity score matching, age, multivessels, CTO, LM, lower LVEF, and higher heart rate were significantly and independently associated with the endpoints of all-cause death/HF. Age, previous history of stroke, multivessels, LM, lower LVEF, higher heart rate, and HbA_1_C were the risk factors for the secondary endpoints of all-cause death/HF/nonfatal MI.

## 4. Discussion

We found that patients with *β*-blockers had more CHD risk factors, lower LVEF, more severe coronary artery disease, and were more likely to use other secondary prevention of coronary heart disease at the discharge of our data. Compared with the no *β*-blockers used group, *β*-blocker therapy at discharge was associated with a similar risk of MACCE during 25 months of median follow-up period. After propensity score analysis, the result was also similar, which was consistent with other observational analyses.

As it is well known, the sympathetic adrenergic nervous system plays a fundamental role in the homeostatic regulation of cardiovascular function. Some diseases presenting decreased myocardial function can elicit activity from the sympathetic nervous system, which leads to the release of catecholamines through the G protein-coupled receptor (GPCR) system and leads to increased mechanical stress on the failing heart and causing harmful electrical and structural events [11, 12]. Moreover, augmented levels of catecholamines can cause myocardial damage via enhanced cardiac oxygen demand and by increasing peroxidative metabolism [12]. *α*_1_-Adrenergic receptors (ARs) and *β*-ARs are the two major categories of myocardial ARs. *α*_1_-AR stimulation can activate the enzyme phospholipase C (PLC). Activation of PLC generates the second messengers, inositol trisphosphate (IP3) and 2-diacylglycerol (DAG), ultimately ending with the release of intracellular calcium, producing positive inotropy (especially in failing myocardium), promotes adaptive hypertrophy, induces ischemic preconditioning, and prevents cardiac myocyte death [13]. Stimulation *β*_1_-ARs and *β*_2_-ARs can activate the stimulatory G protein/adenylyl-cyclase/cAMP/protein kinase A (PKA) signaling pathway, enhancing myocardial contractility and heart rate [11]. In addition, *β*_1_-ARs are associated with extracellular signal-regulated kinases (ERK) 1 and 2, and *β*-blockers can stimulate ERK1 and ERK2, which can produce cardioprotective during ischemia and heart failure [14]. G protein-coupled receptor kinase 2 (GRK2) is the most important isoform related to cardiac physiology. GRK2 appears to regulate cardiomyocyte function in part by controlling *β*_1_-AR in the regulation of cardiac contractility and chronotropic. GRK2 levels may reflect hemodynamic impairment and might have a meaningful prognostic value after myocardial infarction. Furthermore, GRK2 upregulation also affects cardiac metabolism and, in particular, myocardial glucose uptake [11]. Stimulation of *β*-ARs also can affect cytokines. *β*-Adrenoceptor-mediated activation of cAMP-responsive element and activating protein-1 directly contributes to interleukin-6 induction in the failing myocardium, which can prevent decompensation during cardiac overload and attenuates *β*-adrenergic inotropy [15]. Fibroblast growth factor 21 (FGF21) is induced by catecholamine via AMP-activated protein kinase activation in cardiomyocytes and FGF21 in turn activates FGF21 expression and AMPK pathway, having antioxidative and anti-hypertrophic effects [16]. In addition, *β*_2_-ARs also can activate the inhibitory G protein and *β*-arrestins, which have a major role in cardiomyocyte growth and cardiac hypertrophy. A previous study showed that antagonists cannot influence *β*_2_-ARs by combining *β*-arrestins [17]. GRK2 is implicated in the phosphorylation-dependent desensitization of *β*_2_-AR, which results in the dissociation of G protein from the receptor and its subsequent internalization mediated by *β*-arrestins, could be implicated in osteogenic differentiation of vascular smooth muscle cells or pericytes during artery calcification, and will be associated with an increased coronary artery calcification progression [18]. Recently, it has also been found that *β*_3_-ARs exist in the cardiomyocytes. *β*_3_-ARs are typically activated by high concentrations of catecholamines, stimulating nitric oxide synthase, thereby increasing cGMP levels and activating protein kinase G, which has potential negative inotropic effects [11]. Cardiac hypertrophy and heart failure are typically characterized by derangement of *β*-AR signaling and a reduction in the adrenergic reserve of the heart. This is primarily due to the selective downregulation of *β*_1_-AR density at the plasma membrane and by the uncoupling of the remaining *β*_1_-ARs and *β*_2_-ARs from G proteins. The previous study also showed that the elevated sympathetic activity in chronic heart failure cause enhanced GRK2-mediated cardiac *β*_1_-AR and *β*_2_-AR desensitization and *β*_1_-AR downregulation, the compensatory upregulation of *β*_3_-ARs, eventually leading to the progressive loss of the adrenergic, inotropic reserves of the heart, and the deterioration of cardiac function [11].

Therefore, counteracting adrenergic overdrive via *β*-AR antagonists reduces cardiac workload and increases O_2_ sparing in patients with failing hearts. Therefore, the consensus guidelines recommended early use in all UAP and non-ST-segment elevation myocardial fraction patients without contraindications within 24 hours. However, the usage of *β*-blockers can cause glucose and lipid metabolism disorders and is due to the blockade of *β*_2_-AR-dependent insulin release from the pancreatic islets of Langerhans [11, 19]. Selective *β*-blocker usage did not contribute to the glucose metabolism [19]. In addition, animal experiments show that *β*_1_-ARs can mediate vasodilator responses of rat cerebral arteries, implying that *β*-blockers may impair cerebral blood flow under some conditions, inducing ischemic stroke [20]. Thus, it has a good prospect for shifting the paradigm from purely adrenergic blockade to comprehensive adrenergic modulation.

Indeed, current research results about the application are inconsistent. Current advances mostly come from patients with myocardial infarction and heart failure, and it remains unclear whether other CHD patients benefit from *β*-blockers. *β*-blockers are beneficial in STEMI patients if given early PCI and hemodynamically stable, and this effect of *β*-blockers was largely driven by a reduction in ventricular arrhythmias and reinfarction [21, 22], and it may improve survival rate. Tetsuro et al. [23] showed that the use of *β*-blockers in patients with myocardial infarction or HF with reduced left ventricular ejection fraction and DM was associated with a decreased risk of all-cause mortality. However, some studies emphasized that *β*-blockers have no benefit, which has long been reflected in the clinical guidelines, which recommend early use [7]. Meta-analysis revealed that *β*-blockers do not provide any survival benefit in patients with angiographic CHD without a history of myocardial infarction or reduced ejection fraction [24]. Another study showed that *β*-blockers do not decrease the mortality of patients with post-myocardial infarction, especially more than 1 year after myocardial infarction[25]. The genetic variants and race differences are also associated with survival among ACS patients treated with *β*-blockers [26]. Therefore, the current use of *β*-blockers for risk reduction has increasingly come under question [27, 28]. These results are mostly confined to patients with AMI. Therefore, the evidence for using *β*-blockers in current UAP patients is lacking.

In our study, *β*-blockers appear to be frequently utilized (nearly 2 of every 3 patients) in patients with UAP at discharge. About the unadjusted analysis between those who did and did not receive *β*-blockers, the results revealed a large difference in all-cause death/HF, all-cause death/HF/non-fatal MI, and all-cause death/HF/non-fatal MI/non-fatal stroke. But after adjusted analysis, the difference was not observed. After propensity score analysis, *β*-blocker use was associated with a significant increase in the composite endpoint of all-cause death/HF and all-cause death/HF/nonfatal MI in this patient group. Similarly, the difference was not observed after the adjusted analysis. These conditions likely reflect the fact that unadjusted analysis in the observational studies might be influenced by selection bias and some confounding factors. *β*-blockers at discharge were associated with a nonsignificant difference in the risk of all-cause death, cardiac death, and HF, respectively, during the follow-up period.

We did not observe the benefit of *β*-blockers in UAP patients and the reason may be that first the proportion of PCI in all the selected patients was 57.8%, and successful PCI maybe reduces the mobility of recurrence of ischemic heart disease, and it will offset the benefit of *β*-blockers. Second, although *β*-blockers exert their effects by competitively inhibiting catecholamine binding to *β*-receptors [23], patients with UAP may have lower sympathetic excitability than AMI, which leads to hypofunction of *α*_1_-AR and *β*-ARs. The role may be rare. Third, *β*-blockers also had some side effects on both glucose and lipid metabolism that theoretically could increase the risk of cardiovascular disease [29–31]. Fourth, *β*-blockers had differences in hemodynamic effects, which can reduce brachial blood pressure, not central systolic blood pressure [32]. Fifth, the variability of *β*-blocker usage in acute coronary syndrome might be related in part to genetic heterogeneity [26]. Sixth, *β*-blockers may only block *β*_1_-ARs and *β*_2_-ARs, but not block *β*_3_-ARs, producing potential negative inotropic effects. These disadvantages of *β*-blockers may become more evident in normal clinical practice. These may be why the values were not revealed.

To date, several studies have shown that elevated heart rate was associated with an increased risk of long-term mortality after AMI [33, 34]. Our results were consistent with those studies. We observed that heart rate at admission was an independent risk factor for all-cause death/HF, all-cause death/HF/nonfatal MI, and all-cause death/HF/nonfatal MI/nonfatal stroke after multivariate regression analysis, not *β*-blockers. After propensity score matching, this phenomenon also appeared in the secondary composite endpoints of all-cause death/HF and all-cause death/HF/nonfatal MI. The results show that reducing heart rate at admission can benefit in the long-term follow-up period. In other words, using *β*-blockers in UAP patients is suboptimum, but controlling heart rate at admission can reduce MACCE and secondary endpoints.

## 5. Study Limitations

First, this is an observational study performed at a single national center. Second, only patients who survived the hospital stay were included, and the role of in-hospital *β*-blockers was not investigated. In addition, there was no information in this study about rates of discontinuation, duration or doses, and kinds of *β*-blockers after hospital discharge. Last but not least, the low ejection fraction was rarely in our data and therefore not included in our subgroup analysis, and we did not analyze the heart rate at discharge. The result may be partial.

## 6. Conclusion

Among patients who survived hospitalization with UAP, *β*-blocker use was not associated with lower MACCE and other secondary composite endpoints in long-term outcomes. This result adds to the increasing body of evidence that the routine prescription of *β*-blockers might not be indicated in patients with UAP.

## Figures and Tables

**Table 1:** Clinical characteristics of patients in the *β*-blocker and no *β*-blocker groups.

	Before PS match			After PS match		
	*β*-Blocker (-) *N* = 1801	*β*-Blocker (+) *N* = 3790	*P* value	*β*-Blocker (-) *N* = 1786	*β*-Blocker (+) *N* = 1786	*P* value
Age (years)	65.0 ± 9.4	64.4 ± 9.5	0.035	65.0 ± 9.4	65.0 ± 9.3	0.947
Male (%)	1156 (64.2)	2379 (62.8)	0.305	1142 (63.9)	1145 (64.1)	0.917
BMI (kg/m^2^)	25.8 ± 3.4	26.1 ± 3.5	0.004	25.8 ± 3.4	25.7 ± 3.4	0.298
SBP (mmHg)	132 ± 17.2	132.7 ± 17.3	0.144	132.1 ± 17.2	132.0 ± 16.7	0.790
DBP (mmHg)	75.6 ± 11.4	76.8 ± 11.0	0.001	75.8 ± 11.4	76.1 ± 10.9	0.387
Heart rate (bpm)	66 (60,73)	71 (64,78)	<0.001	66 (60,73)	70 (64,78)	<0.001

*Medical history*						
Current smoker (%)	569 (31.6)	1186 (31.3)	0.821	562 (31.5)	548 (30.7)	0.613
Hypertension (%)	1216 (67.5)	2842 (75.0)	<0.001	1216 (68.1)	1226 (68.6)	0.719
DM (%)	703 (39.0)	1616 (42.6)	<0.05	700 (39.2)	712 (39.9)	0.681
Dyslipidemia (%)	878 (48.8)	1909 (50.4)	0.258	873 (48.9)	862 (48.3)	0.713
Stroke (%)	286 (15.9)	625 (16.5)	0.563	285 (16.0)	297 (16.6)	0.587
CHD (%)	816 (45.3)	1994 (52.6)	<0.001	815 (45.6)	821 (46.0)	0.840
OMI (%)	104 (5.8)	333 (8.8)	<0.001	104 (5.8)	104 (5.8)	1.000
CABG (%)	27 (1.5)	97 (2.6)	0.012	27 (1.5)	27 (1.5)	1.000
PCI (%)	235 (13)	696 (18.4)	<0.001	235 (13.2)	229 (12.8)	0.765

*Medication used before admission*						
Antiplatelet agent (%)	669 (37.1)	1646 (43.4)	<0.001	669 (37.5)	678 (38.0)	0.756
ACEI/ARB (%)	697 (38.7)	1483 (39.1)	0.759	697 (39.0)	720 (40.3)	0.431
*β*-Blockers (%)	154 (8.6)	1378 (36.4)	<0.001	154 (8.6)	605 (33.8)	<0.001
Statins (%)	595 (33.0)	1345 (35.5)	0.072	594 (33.3)	601 (33.7)	0.804

Data are presented as mean ± SD, IQR, or *n* (%). BMI, body mass index; SBP, systolic blood pressure; DBP, diastolic blood pressure; DM, diabetes mellitus; CHD, coronary heart disease; OMI, old myocardial infarction; CABG, coronary artery bypass grafting; PCI, percutaneous coronary intervention; ACEI, angiotensin-converting enzyme inhibitor; ARB, angiotensin II receptor blocker.

**Table 2:** Laboratory test results and echocardiographic and angiographic characteristics.

	Before PS match			After PS match		
	*β*-Blocker (-) *N* = 1801	*β*-Blocker (+) *N* = 3790	*P* value	*β*-Blocker (-) *N* = 1786	*β*-Blocker (+) *N* = 1786	*P* value
*Laboratory values*						
WBC (×10^9^/L)	6.1 (5.2, 7.3)	6.4 (5.4, 7.5)	<0.001	6.2 (5.2, 7.3)	6.3 (5.3, 7.4)	0.011
Hemoglobin (g/L)	135.6 ± 15.9	135.4 ± 16.8	0.648	135.6 ± 15.9	135.0 ± 17.3	0.245
PLT (×10^12^/L)	211.0 (179.0, 248.0)	215.0 (180.0, 254.0)	0.021	211.0 (179.0, 249.0)	211.0 (177.0, 250.0)	0.988
HsCRP (mg/L)	1.3 (0.6, 2.5)	1.6 (0.7, 3.3)	<0.001	1.3 (0.6, 2.5)	1.5 (0.6, 3.3)	0.001
FBG (mmol/l)	5.8 (5.0, 7.5)	6.1 (5.1, 8.0)	<0.001	5.8 (5.0, 7.5)	5.9 (5.0, 7.7)	0.432
HbA1C (%)	6.0 (5.6, 6.8)	6.2 (5.7, 7.2)	<0.001	6.0 (5.6, 6.8)	6.1 (5.6, 6.9)	0.294
ALT (U/L)	17.0 (12.0, 24.0)	18.0 (13.0, 26.0)	<0.001	17.0 (12.8, 24.0)	17.0 (13.0, 25.0)	0.211
Creatinine (*μ*mol/L)	75.3 (64.9, 86.1)	75.9 (65.5, 87.9)	0.354	75.2 (64.9, 86.1)	75.4 (65.5, 86.9)	0.318
eGFR (mL/min/1.73 m^2^)	156.6 (124.1, 191.9)	153.3 (122.6, 190.9)	0.084	156.6 (124.0,192.0)	155.0 (124.2,190.5)	0.594
TC (mmol/L)	4.1 (3.5, 4.8)	4.0 (3.4, 4.8)	0.165	4.1 (3.5, 4.8)	4.1 (3.4, 4.8)	0.287
TG (mmol/L)	1.3 (1.0, 1.9)	1.4 (1.0, 2.0)	<0.001	1.3 (1.0, 1.9)	1.3 (1.0, 1.9)	0.734
HDL (mmol/L)	1.1 (0.9, 1.3)	1.1 (0.9, 1.2)	0.197	1.1 (0.9, 1.3)	1.1 (0.9, 1.2)	0.978
LDL (mmol/L)	2.3 (1.8, 2.8)	2.2 (1.8, 2.7)	0.074	2.3 (1.8, 2.8)	2.2 (1.8, 2.7)	0.127

*Echocardiographic values*						
LA (cm)	3.7 ± 0.4	3.7 ± 0.5	0.091	3.7 ± 0.4	3.7 ± 0.5	0.406
LVEDD (cm)	5.0 (4.7, 5.3)	5.0 (4.7, 5.3)	0.912	5.0 (4.7, 5.3)	5.0 (4.7, 5.3)	0.330
LVESD (cm)	3.1 (2.9, 3.4)	3.1 (2.9, 3.4)	0.076	3.1 (2.9, 3.4)	3.1 (2.9, 3.4)	0.086
LVEF (%)	0.67 (0.64, 0.71)	0.67 (0.63, 0.70)	0.026	0.67 (0.64, 0.71)	0.67 (0.63, 0.70)	0.129
LVEF (40–49) (%)	48 (2.7)	186 (4.9)	<0.001	48 (2.7)	69 (3.9)	0.048
LVEF (<40) (%)	10 (0.6)	58 (1.5)	<0.001	10 (0.6)	24 (1.3)	0.039
LVFS (%)	0.38 (0.35, 0.40)	0.37 (0.34, 0.40)	0.025	0.38 (0.35, 0.40)	0.37 (0.35,0.40)	0.138

*Angiography values*						
LM (%)	164 (9.1)	402 (10.6)	0.082	161 (9.0)	183 (10.2)	0.212
Multivessels (%)	1379 (76.6)	3155 (83.2)	<0.001	1368 (76.6)	1446 (81.0)	0.001
CTO (%)	140 (7.8)	404 (10.7)	0.001	140 (7.8)	181 (10.1)	0.016
PCI (%)	957 (53.1)	2277 (60.1)	<0.001	950 (53.2)	1087 (60.9)	<0.001

*Medication used at discharge*						
Antiplatelet agent (%)	1670 (92.7)	3727 (98.3)	<0.001	1657 (92.8)	1757 (98.4)	<0.001
ACEI/ARB (%)	798 (44.3)	1984 (52.3)	<0.001	796 (44.6)	920 (51.5)	<0.001
Statins (%)	1582 (87.8)	3508 (92.6)	<0.001	1570 (87.9)	1672 (93.6)	<0.001

Data are presented as mean ± SD, IQR, or *n* (%). WBC, white blood cells; PLT, platelet count; hsCRP, hypersensitivity C-reactive protein; eGFR, estimated glomerular filtration rate; FBG, fasting blood glucose; HbA_I_C, glycosylated hemoglobin; ALT, alanine aminotransferase; TC, total cholesterol; TG, triglyceride; LDL-C, low-density lipoprotein cholesterol; HDL-C, high-density lipoprotein cholesterol; LVEDD, left ventricular end-diastolic dimension; LVESD, left ventricular end-systolic dimension; LVEF, left ventricular ejection fraction; LVFS, left ventricular fraction shortening; LM, left main trunk; CTO, chronic total occlusions; PCI, percutaneous coronary intervention; ACEI, angiotensin-converting enzyme inhibitor; ARB, angiotensin II receptor blocker.

**Table 3:** Major adverse cardiac and cerebral events in patients with UAP in the *β*-blocker and no *β*-blocker groups.

	Before PS match			After PS match		
	*β*-Blocker (-) *N* = 1801	*β*-Blocker (+) *N* = 3790	*P* value	*β*-Blocker (-) *N* = 1786	*β*-Blocker (+) *N* = 1786	*P* value
Event						
*Composite MACCE*						
No. of patients	420	1005		417	463	
Event rate (%)	23.3	26.5	0.010	23.3	25.9	0.074
Unadjusted HR (95% CI)	1.00	1.08 (0.96, 1.21)	0.212	1.00	1.04 (0.91, 1.19)	0.545
Adjusted HR (95% CI)	1.00	0.98 (0.87, 1.10)	0.725	1.00	0.98 (0.86, 1.13)	0.812

*All-cause death*						
No. of patients	49	142		49	70	
Event rate (%)	2.7	3.7	0.048	2.7	3.9	0.050
Unadjusted HR (95% CI)	1.00	1.29 (0.93, 1.78)	0.129	1.00	1.34 (0.93, 1.93)	0.114
Adjusted HR (95% CI)	1.00	1.05 (0.75, 1.47)	0.769	1.00	1.30 (0.90, 1.89)	0.163

*Cardiac death*						
No. of patients	17	53		17	26	
Event rate (%)	0.9	1.4	0.153	1.0	1.5	0.167
Unadjusted HR (95% CI)	1.00	1.38 (0.80, 2.38)	0.248	1.00	1.43 (0.78, 2.63)	0.253
Adjusted HR (95% CI)	1.00	0.98 (0.56, 1.71)	0.930	1.00	1.27 (0.68, 2.39)	0.454

*Nonfatal MI*						
No. of patients	32	72		32	35	
Event rate (%)	1.8	1.9	0.751	1.8	2.0	0.711
Unadjusted HR (95% CI)	1.00	1.01 (0.67, 1.53)	0.958	1.00	1.04 (0.65, 1.69)	0.864
Adjusted HR (95% CI)	1.00	0.88 (0.57, 1.35)	0.558	1.00	1.06 (0.65, 1.72)	0.814

*Nonfatal stroke*						
No. of patients	14	41		14	17	
Event rate (%)	0.8	1.1	0.281	0.8	1.0	0.588
Unadjusted HR (95% CI)	1.00	1.28 (0.70, 2.35)	0.423	1.00	1.12 (0.55, 2.27)	0.757
Adjusted HR (95% CI)	1.00	1.04 (0.56, 1.94)	0.891	1.00	1.13 (0.56, 2.31)	0.732

*Heart failure*						
No. of patients	25	82		18	29	
Event rate (%)	1.4	2.2	0.048	1.0	1.6	0.106
Unadjusted HR (95% CI)	1.00	1.48 (0.95, 2.32)	0.085	1.00	1.55 (0.56, 2.78)	0.147
Adjusted HR (95% CI)	1.00	1.20 (0.78, 1.89)	0.445	1.00	1.28 (0.71, 2.32)	0.409

*Cardiac rehospitalization*						
No. of patients	375	874		372	400	
Event rate (%)	20.8	23.1	0.060	20.8	22.4	0.255
Unadjusted HR (95% CI)	1.00	1.05 (0.93, 1.18)	0.456	1.00	1.02 (0.88, 1.17)	0.803
Adjusted HR (95% CI)	1.00	0.96 (0.85, 1.09)	0.554	1.00	0.99 (0.86, 1.14)	0.889

Data are presented as number or HR (95% CI). MACCE, major adverse cardiac and cerebral events; MI, myocardial infarction.

**Table 4:** Secondary endpoints in patients with UAP in the *β*-blocker and no *β*-blocker groups.

	Before PS match			After PS match		
	*β*-Blocker (-) *N* = 1801	*β*-Blocker (+) *N* = 3790	*P* value	*β*-Blocker (-) *N* = 1786	*β*-Blocker (+) *N* = 1786	*P* value
Event						
*All-cause death or heart failure*						
No. of patients	68	214		67	99	
Event rate (%)	3.8	5.6	0.003	3.8	5.5	0.011
Unadjusted HR (95% CI)	1.00	1.41 (1.07, 1.85)	0.013	1.00	1.40 (1.03, 1.91)	0.033
Adjusted HR (95% CI)	1.00	1.16 (0.88,1.53)	0.296	1.00	1.20 (0.88, 1.65)	0.256

*All-cause death, heart failure, or nonfatal MI*						
No. of patients	94	272		93	128	
Event rate (%)	5.2	7.2	0.006	5.2	7.2	0.015
Unadjusted HR (95% CI)	1.00	1.30 (1.03, 1.65)	0.027	1.00	1.31 (1.01, 1.71)	0.046
Adjusted HR (95% CI)	1.00	1.08 (0.85, 1.38)	0.520	1.00	1.17 (0.89,1.53)	0.267

*All-cause death, heart failure, nonfatal MI, or nonfatal stroke*						
No. of patients	108	305		107	141	
Event rate (%)	6.0	8.0	0.006	6.0	7.9	0.025
Unadjusted HR (95% CI)	1.00	1.27 (1.02, 1.58)	0.035	1.00	1.25 (0.97, 1.61)	0.082
Adjusted HR (95% CI)	1.00	1.04 (0.83, 1.31)	0.712	1.00	1.11 (0.86, 1.44)	0.410

Data are presented as number or HR (95% CI). MI, myocardial infarction.

**Table 5:** Multivariate Cox regression analysis of secondary endpoints.

	Univariate		Multivariate					
	HR (95% CI)	*P* value	A		B		C	
			HR (95% CI)	*P* value	HR (95% CI)	*P* value	HR (95% CI)	*P* value
Age (years)	1.07 (1.06, 1.08)	<0.001	1.07 (1.05, 1.08)	<0.001	1.05 (1.04, 1.07)	<0.001	1.05 (1.04, 1.06)	<0.001
Male (%)	0.89 (0.68, 1.09)	0.205						
Stroke (%)	1.86 (1.43, 2.43)	<0.001	1.44 (1.10, 1.88)	0.008	1.38 (1.09, 1.76)	0.008	1.3861.08, 1.70)	0.009
Heart rate (bpm)	1.02 (1.01, 1.03)	<0.001	1.02 (1.01, 1.03)	<0.001	1.01 (1.00, 1.02)	0.002	1.01 (1.00, 1.02)	<0.001
hsCRP (mg/L)	1.03 (1.02, 1.05)	<0.001	1.02 (1.00, 1.03)	0.027	1.02 (1.00, 1.03)	0.034	1.02 (1.00, 1.03)	0.026
HbA_1_C (%)	1.14 (1.06, 1.23)	<0.001	1.09 (1.01, 1.19)	0.032	1.15 (1.07, 1.23)	<0.001	1.15 (1.07, 1.22)	<0.001
LVEF ≥ 50%	Ref		Ref		Ref		Ref	
40% ≤ LVEF < 50%	2.63 (1.67, 4.15)	<0.001	1.94 (1.21, 3.10)	0.006	1.69 (1.10, 2.61)	0.017	1.78 (1.19, 2.66)	0.005
LVEF < 40%	6.41 (3.92, 10.49)	<0.001	6.24 (3.76, 10.35)	<0.001	4.42 (2.69, 7.29)	<0.001	4.25 (2.65, 6.81)	<0.001
LM (%)	2.06 (1.53, 2.78)	<0.001	1.47 (1.08, 2.00)	0.016	1.39 (1.06, 1.84)	0.019		
Multivessels (%)	3.58 (2.19, 5.85)	<0.001	2.47 (1.50, 4.08)	<0.001	2.30 (1.51, 3.52)	<0.001	2.28 (1.56, 3.37)	<0.001
CTO (%)	2.03 (1.50, 2.76)	<0.001						
*β*-Blockers at discharge (%)	1.41 (1.07, 1.85)	0.013						

A, present all-cause death/HF; B, present all-cause death/HF/nonfatal MI; C, present all-cause death/HF/nonfatal MI/nonfatal stroke. MI, myocardial infarction; HbA_I_C, glycosylated hemoglobin; hsCRP, hypersensitivity C-reactive protein; LVEF, left ventricular ejection fraction; LM, left main trunk; CTO, chronic total occlusions.

**Table 6:** Multivariate Cox regression analysis of secondary endpoints in propensity score matching patients.

	Univariate		Multivariate			
	HR (95% CI)	*P* value	A		B	
			HR (95% CI)	*P* value	HR (95% CI)	*P* value
Age (years)	1.08 (1.06, 1.10)	<0.001	1.07 (1.05, 1.09)	<0.001	1.06 (1.04, 1.07)	<0.001
Male (%)	0.85 (0.63, 1.17)	0.320				
Stroke (%)	1.90 (1.35, 2.68)	<0.001			1.47 (1.08, 1.98)	0.014
Heart rate (bpm)	1.02 (1.01, 1.03)	<0.001	1.02 (1.01, 1.03)	0.001	1.01 (1.00, 1.02)	0.030
hsCRP (mg/L)	1.02 (1.00, 1.05)	0.031				
HbA_1_C (%)	1.15 (1.04, 1.28)	0.008			1.19 (1.08, 1.30)	<0.001
LVEF ≥ 50%	Ref		Ref		Ref	
40% ≤ LVEF < 50%	2.93 (1.59, 5.41)	0.001	2.23 (1.19, 4.16)	0.012	1.89 (1.07, 3.34)	0.029
LVEF < 40%	1.30 (0.32, 5.24)	0.714	1.19 (0.29, 4.87)	0.814	0.87 (0.21, 3.54)	0.845
LM (%)	2.47 (1.70, 3.61)	<0.001	1.62 (1.09, 2.39)	0.016	1.52 (1.08, 2.16)	0.017
Multivessels (%)	3.78 (2.05, 6.97)	<0.001	260 (1.39, 4.85)	0.003	2.22 (1.34, 3.69)	0.002
CTO (%)	2.42 (1.64, 3.58)	<0.001	1.71 (1.13, 2.57)	0.010		
*β*-Blockers at discharge (%)	1.40 (1.03, 1.91)	0.033				

A, present all-cause death/HF; B, present all-cause death/HF/nonfatal MI. MI, myocardial infarction; HbA_I_C, glycosylated hemoglobin; hsCRP, hypersensitivity C-reactive protein; LVEF, left ventricular ejection fraction; LM, left main trunk; CTO, chronic total occlusions.

## Data Availability

The datasets used and/or analyzed during this study are available from the corresponding author on reasonable request.

## Abbreviations

BMI: Body mass index
SBP: Systolic blood pressure
DBP: Diastolic blood pressure
MI: Myocardial infarction
CHD: Coronary heart disease
OMI: Old myocardial infarction
DM: Diabetes mellitus
WBC: White blood cells
hsCRP: Hypersensitivity C-reactive protein
eGFR: Estimated glomerular filtration rate
FBG: Fasting blood glucose
TC: Total cholesterol
TG: Triglyceride
LDL-C: Low-density lipoprotein cholesterol
HDL-C: High-density lipoprotein cholesterol
LVEDD: Left ventricular end-diastolic dimension
LVESD: Left ventricular end-systolic dimension
LVEF: Left ventricular ejection fraction
LVFS: Left ventricular fraction shortening
LM: Left main trunk
PCI: Percutaneous coronary intervention
CABG: Coronary artery bypass grafting
ACEI: Angiotensin-converting enzyme inhibitor
ARB: Angiotensin II receptor blocker
PLT: Platelet count
HbA_I_C: Glycosylated hemoglobin
ALT: Alanine aminotransferase
CTO: Chronic total occlusions
ACEI: Angiotensin-converting enzyme inhibitor
ARB: Angiotensin II receptor blocker
MACCE: Major adverse cardiac and cerebral events.
